# Epidemic thresholds across sexual behavior groups in the UK Biobank

**DOI:** 10.3389/frph.2026.1790557

**Published:** 2026-03-25

**Authors:** Maxence Arutkin, Alexandre Vallée

**Affiliations:** 1 School of Chemistry, Tel Aviv University, Tel Aviv, Israel; 2 Department of Epidemiology and Public Health, Foch Hospital, Suresnes, France; 3 IHU FOReSIGHT, Paris, France

**Keywords:** degree heterogeneity, epidemic threshold, heavy-tailed distributions, sexual networks, UK Biobank

## Abstract

**Background:**

Sexual partnership heterogeneity strongly conditions the epidemic potential of sexually transmitted infections (STIs), while bisexual partnerships can connect otherwise distinct sexual networks and enable spillover across populations. An actionable summary of this structure is the configuration-model critical bond transmissibility $T_{c}$, which links right-tail heterogeneity in partner counts to a static percolation threshold (interpreted here as a structural susceptibility index under a lifetime-partner proxy).

**Methods:**

We analyzed the lifetime number of sexual partners of 405,740 UK Biobank participants (aged 40–69), stratified into men with male partners (MSM), men with female-only partners (MSW), women with male-only partners (WSM), and women with female partners (WSW). Candidate heavy-tail models were fitted to the fixed top-50% tail in each stratum using discrete maximum likelihood on identical integer support. Robustness to digit preference was assessed under prespecified RAW/EXCLUDE/EM-like modes. Epidemic thresholds were estimated from empirical moments, and a two-group next-generation matrix was used to derive minimal bisexual-bridging conditions for cross-group transmission.

**Results:**

In non-MSM strata, the tails were heavy but finite, with discretized lognormal or stretched-exponential models outperforming a pure power law and strictly positive thresholds (WSM $T_{c}\approx 0.055$; MSW $T_{c}\approx 0.005$; WSW $T_{c}\approx 0.006$). In MSM, extreme right-tail heterogeneity yielded a near-zero threshold ($T_{c}\approx 3\times 10^{-4}$), indicating high structural susceptibility to sustained spread. Threshold ordering was robust across sensitivity modes. The bridging analysis showed that modest cross-group mixing (illustratively ∼5%–7%) can sustain transmission under plausible within-group controls.

**Conclusion:**

Sexual network tail structure differs sharply by sexual behavior stratum and translates into large, interpretable gaps in epidemic thresholds. These results support targeted, tail-sensitive STI prevention and explicit control of bridging pathways.

## 1 Introduction

Sexual partnership heterogeneity is a primary determinant of sexually transmitted infection (STI) spread: a small fraction of individuals with many partners can disproportionately sustain transmission, and bisexual partnerships can connect otherwise distinct sexual networks and enable spillover across populations (1, 2). For public health, the practical question is not whether contact distributions “look heavy-tailed,” but how strongly their right tails amplify epidemic potential and how this amplification differs across sexual behavior strata that are targeted by prevention programs. Quantifying these differences is essential for translating behavioral heterogeneity into actionable thresholds for interventions such as testing frequency, condom promotion, pre-exposure prophylaxis (PrEP), and rapid treatment.

A principled way to link partnership heterogeneity to epidemic potential is through the configuration model threshold for bond transmissibility, which depends on the ratio of the second to the first moment of the degree distribution. Specifically, for a degree distribution P(K), the critical transmissibility is

$$T_{c}=\frac{E[K]}{E[K^{2}]-E[K]},$$ (1)

so heavier right tails (larger $E[K^{2}]$ relative to E[K]) imply smaller $T_{c}$ and greater structural vulnerability to sustained spread (3, 4). In practice, this provides an interpretable bridge between descriptive sexual behavior data and prevention: lower thresholds indicate that even low per-partnership transmission probabilities may be sufficient for persistence, whereas higher thresholds suggest that control is feasible with moderate reductions in effective transmissibility. Because K here is a lifetime partner count proxy rather than a time-window degree, $T_{c}$ should be interpreted as a configuration model percolation/susceptibility index (a static summary of heterogeneity) rather than a literal STI epidemic threshold; nonetheless, such static thresholds abstract from timing, concurrency, and turnover and offer a transparent first-order summary of how right-tail heterogeneity conditions epidemic potential and how prevention leverage may differ across groups (5–9).

Reliable inference on the right tail of partnership counts is technically challenging. Lifetime partner counts are discrete, often exhibit digit preference (heaping at salient integers), and are frequently analyzed using continuous approximations or *post-hoc* tail choices that can bias tail characterization and, in turn, threshold estimates (10–15). Moreover, several heavy-tailed parametric families can produce similar log–log appearances while implying different tail behavior and different sensitivities of E[K] and $E[K^{2}]$ to extreme observations (16–21). From an epidemiological standpoint, a useful tail model must therefore be (i) fitted on the correct discrete support, (ii) compared like-for-like across candidate families, and (iii) robust to heaping and extreme records—because these features directly affect moment-based thresholds.

The UK Biobank (UKB) provides the lifetime number of sexual partners for approximately $4.06\times 10^{5}$ participants, together with indicators of same-sex behavior, enabling large-scale stratified analyses across both sexes and orientations. We prespecified four strata: men with male partners (MSM), men with female-only partners (MSW), women with female partners (WSW), and women with male-only partners (WSM). Compared with nationally representative sexual behavior surveys (e.g., Natsal-3, ∼15,000 adults), the UKB offers one to two orders of magnitude more observations for estimating tail behavior and moments within strata, while covering both sexes and same-sex behavior in a single cohort (22–24). Because the UKB is a volunteer cohort, we focus on internal, stratified contrasts in tail structure and threshold magnitudes rather than population prevalence estimates.

In this study, we develop a discrete, fixed-support inference pipeline designed for count data: we fit candidate heavy-tail models using discrete maximum likelihood on identical integer support (avoiding continuous approximations and confounding from $x_{\min}$ search), evaluate sensitivity to digit preference through prespecified heaping corrections, and translate observed heterogeneity into empirical threshold estimates. In addition, because bisexual partnerships can connect strata and shape spillover risk, we provide a transparent two-group bridge analysis to quantify the minimum cross-group mixing required for sustained transmission under plausible within-group control.

Accordingly, our aims were to (i) characterize the right-tail form of lifetime partnership distributions within each sexual behavior stratum using discrete, fixed-support inference; (ii) estimate stratum-specific empirical epidemic thresholds $T_{c}$ as finite population functionals of observed moments; and (iii) quantify conditions under which bisexual bridging could sustain cross-group transmission. By linking distributional tail properties to interpretable threshold gaps across strata, our results provide a public health framing of sexual network structure that can inform targeted STI prevention strategies.

## 2 Methods

### 2.1 Data source and design

We performed a cross-sectional analysis of UKB baseline questionnaire data (recruitment 2006–2010) focusing on the lifetime number of sexual partners (Field 2149) in participants aged 40–69. The UKB operates under central ethics approval with written informed consent. This study was conducted using data from the UK Biobank under Application ID 101667. The UK Biobank has obtained written informed consent from all participants at enrolment, and holds overarching ethical approval from the North West Multi-Centre Research Ethics Committee (protocol code: 21/NW/0157, date of approval: 21 June 2021). Only fully anonymized and deidentified data were provided to the research team. No minors were included in the cohort (participants were aged 40–69 at recruitment). All analyses were performed in accordance with the UK Biobank’s Data Access Policy and did not require additional consent or ethical approval. As UKB is not sampling representative, we emphasized internal, stratified contrasts rather than population prevalence (Supplementary Material S1.1) (22–25).

### 2.2 Participants and strata

Records with non-missing lifetime partners (Field 2149), sex (Field 31), the ever same-sex indicator (Field 2159), and the lifetime number of same-sex partners (Field 3669) were retained under prespecified cleaning rules (Supplementary Material S1.2). Because Field 3669 is conditionally asked, missing values when Field 2159 = 1 may reflect non-response or routing; we therefore imputed Field 3669 to 1 in those participants as a conservative lower bound and assessed robustness in sensitivity analyses. Four behavioral strata were defined: men reporting at least one male partner (MSM; may also report female partners), men reporting female-only partners (MSW), women reporting at least one female partner (WSW; may also report male partners), and women reporting male-only partners (WSM). The final sample sizes were WSM n=215,734, MSW n=177,679, MSM n=6,428, and WSW n=5,899 (total ≈ 405,740). Moments were computed on the sexually active subgraph (K>0) unless stated otherwise. Per-stratum N used under each heaping mode are reported in Tables 1, 3.

**Table 1:** Fixed-support (top 50%) discrete PL fits by stratum and mode.

Stratum	Mode	$x_{\min}$	$n_{\text{tail}}$	${\mathrm{KS}}_{\text{PL}}$	$\hat{\alpha }$	99% CI (α)
MSM	RAW	12	3,236	0.1309	1.7568	[1.7272, 1.7883]
MSM	EXCLUDE	8	2,298	0.1316	1.7305	[1.6977, 1.7651]
MSM	EM	12	3,236	0.0835	1.7597	[1.7298, 1.7910]
MSW	RAW	4	94,919	0.0790	2.0155	[2.0089, 2.0220]
MSW	EXCLUDE	3	80,426	0.1201	1.9592	[1.9524, 1.9660]
MSW	EM	4	94,919	0.1446	2.0199	[2.0133, 2.0267]
WSW	RAW	8	3,214	0.1148	2.1527	[2.1136, 2.1931]
WSW	EXCLUDE	7	2,347	0.1432	2.0694	[2.0287, 2.1128]
WSW	EM	8	3,214	0.1035	2.1558	[2.1165, 2.1968]
WSM	RAW	3	111,407	0.0979	2.1730	[2.1665, 2.1797]
WSM	EXCLUDE	2	112,509	0.1088	2.0015	[1.9962, 2.0067]
WSM	EM	3	111,407	0.1273	2.1784	[2.1719, 2.1852]

KS is the one-sample KS statistic for PL on the fixed support. The PL exponent α has a 99% CI from a fixed-tail bootstrap (B=50,000).

### 2.3 Reporting bias (digit preference)

To address heaping at salient integers (5, 10, 20, 50, 100, …), we ran the full pipeline under three prespecified modes: RAW (unaltered counts), EXCLUDE (drop observations at a fixed multiplicative heap grid), and EM-like redistribution (deterministic, mass-conserving reallocation within a local window). The same downstream fitting and resampling procedures were applied to each mode (Supplementary Material S1.2).

### 2.4 Tail definition and candidate models

We fixed the tail to the top 50% per stratum (minimum $n_{\text{tail}}\geq 200$) and fitted discrete power law (PL), truncated power law (TPL), lognormal (LN), stretched exponential (SE), and exponential (EXP) on identical support; implementation details are in Supplementary Material S1.1.

### 2.5 Calibration and model selection

Calibration and pairwise model selection [bootstrap goodness of fit (GoF), Vuong tests, Δ Corrected Akaike Information Criterion (AICc)] are detailed in Supplementary Material S1.3.

### 2.6 Moments and epidemic thresholds

The configuration model’s first and second moments were estimated empirically, and the critical bond transmissibility (denoted $T_{c}$) was computed directly from these finite population functionals (Equation 1). Mapping from per-act transmission to per-partnership probability was used solely to translate thresholds into coverage targets for interpretation (Supplementary Material S7; see also Supplementary Material S1.6).

### 2.7 Two-group bridge analysis

Cross-group transmission was analyzed with a two-group next-generation matrix (MSM ↔ WSM). Bridging was mediated by bisexually active men within the MSM stratum who reported female partners, creating MSM–WSM cross-edges. We treated the cross-group mixing parameters as illustrative under degree-independent mixing; in general, edge-balance constraints link the cross-edge fractions when counted from each group. Formal conditions are detailed in Supplementary Material S1.7; full derivation in Supplementary Material S8.

### 2.8 Prespecification, computation, and sensitivity

All hyperparameters (tail keep 50%, $n_{\text{tail}}\geq 200$, bootstrap size 50,000, decision threshold τ=0.10, EM window ±10%) were fixed *a priori*. Computation used the powerlaw package (43) for discrete fits and bootstraps, multiprocessing with single-threaded basic linear algebra subprograms (BLAS), and fixed seeds. Sensitivity analyses included heaping modes, exact perturbations of extreme records, and finite population domain checks (Supplementary Material S1.2, S1.4, S1.8).

## 3 Results

**Figure 1:**
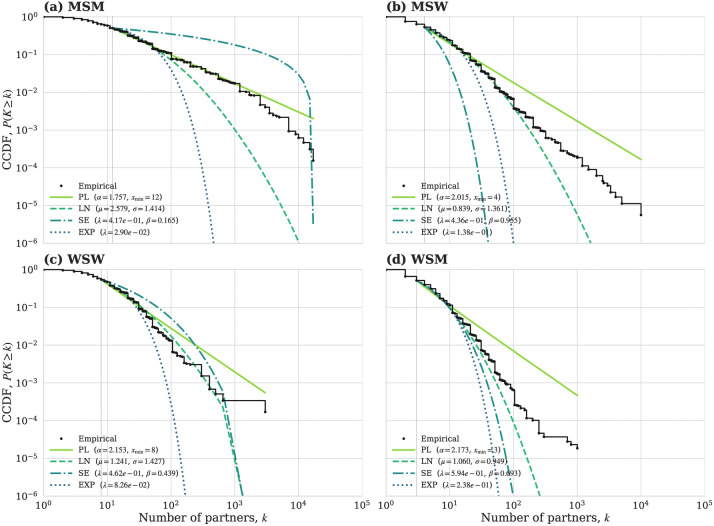
Tail diagnostics for lifetime partner counts by stratum (raw data). Empirical complementary cumulative distribution functions (CCDFs) of the lifetime number of sexual partners K are shown as black dots on log–log axes for four strata: MSM (men who have sex with men), MSW (men who have sex with women), WSW (women who have sex with women), and WSM (women who have sex with men). Colored curves overlay the maximum-likelihood *discrete* tail fits computed for $k\geq x_{\min}$ under four candidate families: power law (PL), lognormal (LN), stretched exponential (SE), and exponential (EXP). The cutoff $x_{\min}$ (here fixed to the stratum-specific median, i.e., a “top–50% tail”), and the fitted parameters are reported in each panel legend. Deviations between fitted curves and the empirical CCDF are most apparent in the far tail and indicate over- or under-prediction of extreme partner counts.

**Reading guide.** Throughout, our tail-fitting diagnostics follow the standard framework for heavy-tail inference introduced by Clauset, Shalizi, and Newman (10) (discrete maximum likelihood, goodness-of-fit by parametric bootstrap, and like-for-like model comparison on a common support). In Figure 1, a power-law tail appears approximately as a straight line on log–log axes; a systematic downward curvature indicates a lighter-than-power-law tail.

### 3.1 Primary tail fits (fixed top–50% tail)

For each stratum and heaping mode, we fixed the tail at the upper 50% of the observed support ($x_{\min}=\lceil {\mathrm{quantile}}_{0.5}(K)\rceil$) and fitted discrete models on the identical support $\left\{k\geq x_{\min}\right\}$. Table 1 reports power–law (PL) fit statistics and 99% CIs for α from the tail–fixed bootstrap with B=50,000.

#### 3.1.1 Power–law goodness–fit (parametric bootstrap, B=50,000, 99% Clopper–Pearson)

Table 2 summarizes the PL GoF decisions at the prespecified threshold τ=0.10. MSM is rejected in all modes (upper CP bound $\approx 1.06\times 10^{-4}$). In contrast, PL is accepted for MSW–RAW and WSM–RAW (lower CP bound ≈0.99985). All other strata–modes are rejected.

**Table 2:** PL GoF on the fixed top–50% tail.

Stratum	Mode	B	Hits	$\hat{p}$	99% CI	Decision
MSM	RAW	50,000	0	0.00000	[0.00000, 0.000106]	Reject
MSM	EXCLUDE	50,000	0	0.00000	[0.00000, 0.000106]	Reject
MSM	EM	50,000	0	0.00000	[0.00000, 0.000106]	Reject
MSW	RAW	50,000	50,000	1.00000	[0.99985, 1.00000]	Accept
MSW	EXCLUDE	50,000	0	0.00000	[0.00000, 0.000106]	Reject
MSW	EM	50,000	0	0.00000	[0.00000, 0.000106]	Reject
WSW	RAW	50,000	0	0.00000	[0.00000, 0.000106]	Reject
WSW	EXCLUDE	50,000	0	0.00000	[0.00000, 0.000106]	Reject
WSW	EM	50,000	0	0.00000	[0.00000, 0.000106]	Reject
WSM	RAW	50,000	49,999	0.99998	[0.99985, 1.00000]	Accept
WSM	EXCLUDE	50,000	0	0.00000	[0.00000, 0.000106]	Reject
WSM	EM	50,000	0	0.00000	[0.00000, 0.000106]	Reject

We report B, hits, $\hat{p}$, 99% Clopper–Pearson interval, and the decision at τ=0.10.

#### 3.1.2 Pairwise model selection on identical support

On the same fixed tail, we compared each alternative to PL using Vuong’s Z (non-nested) and $\Delta \mathrm{AICc}={\mathrm{AICc}}_{\text{alt}}-{\mathrm{AICc}}_{\text{PL}}$. For RAW:

- **MSM**: LN, SE, and EXP are all decisively worse than PL (e.g., LN: Z=−5.864, ΔAICc=+414.30); however PL itself is rejected by GoF. TPL was numerically degenerate on this fixed support.
- **MSW**: LN overwhelmingly dominates PL (Z=+42.07; ΔAICc=−6,572.55). SE and EXP are worse than PL.
- **WSW**: Both LN and SE dominate PL; SE is marginally best (LN: Z=+9.945, ΔAICc=−236.64; SE: Z=+5.356, ΔAICc=−242.79).
- **WSM**: LN decisively dominates PL (Z=+53.21; ΔAICc=−11,365.74); SE is also better than PL but far weaker (ΔAICc=−1,344.32).

(The same qualitative ordering holds under EXCLUDE and EM. On the fixed top–50% support the truncated power law collapsed to the PL boundary ($\hat{\lambda }\to 0$) in all strata and modes (12/12), so we report those rows for completeness but do not interpret them.) Complete RAW-mode pairwise statistics (common support $x_{\min}$/$n_{\text{comp}}$, Vuong Z, and ΔAICc) are reported in Supplementary Table S1. Figure 1 visualizes these fixed-support CCDFs and discrete fits (PL, LN, SE, and EXP) on the identical top-50% tail per stratum.

### 3.2 Sensitivity to digit preference (RAW/EXCLUDE/EM)

GoF and model ranking are robust to heaping corrections. MSM is rejected under all modes; MSW and WSM accept PL only in RAW yet are still decisively better fit by finite-variance alternatives on the same support (LN ≫ PL). WSW tails remain modest in size; SE slightly outperforms LN, but both beat PL.

### 3.3 Empirical configuration–model moments and thresholds

We report empirical moments and thresholds (finite population functionals) per stratum and mode. Table 3 shows the results. MSM exhibits a near-zero threshold ($∼3.4\times 10^{-4}$); WSM is highest ($∼5.5\times 10^{-2}$); MSW and WSW are intermediate.

**Table 3:** Empirical moments and thresholds by stratum and mode.

Stratum	Mode	Method	E[K]	$E[K^{2}]$	$T_{c}$	κ
MSM	RAW	Empirical	78.026447	228,398.448662	0.000342	2,926.192741
MSM	EXCLUDE	Empirical	65.853769	265,933.242605	0.000248	4,037.238757
MSM	EM	Empirical	75.986621	218,759.735221	0.000347	2,877.924372
MSW	RAW	Empirical	8.584166	1,680.766202	0.005134	194.798431
MSW	EXCLUDE	Empirical	6.986139	837.678500	0.008410	118.905783
MSW	EM	Empirical	8.553526	1,515.195200	0.005677	176.142753
WSW	RAW	Empirical	15.185116	2,274.597559	0.006721	148.791252
WSW	EXCLUDE	Empirical	14.375908	2,787.069604	0.005185	192.870855
WSW	EM	Empirical	15.143923	2,257.577216	0.006753	148.074798
WSM	RAW	Empirical	4.518741	87.207913	0.054647	18.299163
WSM	EXCLUDE	Empirical	3.760017	53.367205	0.075796	13.193343
WSM	EM	Empirical	4.514212	83.524725	0.057134	17.502615

### 3.4 Diagnostics and ordering of tail heaviness

Across strata, log–log CCDFs (Figure 1) show systematic curvature relative to a straight PL: empirical/theoretical PL ratios fall below 1 in the far tail for MSW/WSW/WSM (PL too heavy), whereas in MSM, the mid tail lies below PL, but the far tail exceeds LN/SE (alternatives too light).

In MSM (top left), the pure PL (α≈1.76) overpredicts the midtail (line above points for $k∼10^{2}$–$10^{3}$), yet the alternatives (LN/SE/EXP) underpredict the far tail (lines below points), explaining why PL fails the bootstrap GoF while still outperforming LN/SE/EXP in pairwise likelihood on this fixed support. This geometry is consistent with a PL-like head/mid tail with ultimate truncation or mixture, yielding a near-zero empirical threshold ($T_{c}\approx 3.4\times 10^{-4}$).

For MSW and WSM (top right, bottom right), PL sits above the empirical CCDF in the far tail (too heavy), whereas the discretized lognormal closely follows the staircase across the fitted range; this matches the formal selection where LN decisively dominates PL (large negative ΔAICc, Vuong Z>0) despite PL GoF acceptance in RAW. The implied finite variance aligns with strictly positive $T_{c}$ ($\mathrm{MSW}∼5.1\times 10^{-3}$; $\mathrm{WSM}∼5.5\times 10^{-2}$).

WSW (bottom left) shows a smaller, steeper tail; stretched exponential marginally outperforms LN while both beat PL, again indicating finite variance. Overall, the visual ordering—MSM ≪ (MSW, WSW) ≪ WSM in heaviness—is congruent with the moment-based thresholds and with the bridge analysis that follows.

Ordering by epidemic threshold (heaviness) is stable across modes: MSM (smallest $T_{c}$) ≪ (MSW, WSW) ≪ WSM.

## 4 Discussion

Our analysis of lifetime sexual partnership distributions in the UK Biobank offers three substantive contributions to epidemiology and public health. First, we show that outside MSM, the distributions are heavy-tailed yet have finite variance, yielding strictly positive epidemic thresholds. Second, in MSM, the tail remains extremely heavy, with an empirical threshold close to zero, consistent with efficient transmission of low-transmissibility infections. Third, we provide an explicit quantitative framework for bisexual bridging, demonstrating that small fractions of cross-group connections can determine whether infections spill over from MSM to heterosexual populations.

### 4.1 Interpretation of thresholds in relation to STI epidemiology

The empirical thresholds derived here provide a lens through which to interpret observed epidemiological patterns. In WSM, the thresholds were high (≈0.05), suggesting that, under a static configuration model approximation based on lifetime partner counts, sustained spread in this stratum would require comparatively higher effective per-partnership transmissibility (e.g., as for bacterial STIs such as *Chlamydia trachomatis* or *Neisseria gonorrhoeae*). This aligns with their high incidence in general populations (26, 27). Conversely, HIV transmission probabilities per heterosexual partnership are generally in the order of $10^{-3}$–$10^{-2}$, well below this threshold, consistent with the limited heterosexual spread of HIV in the UK and with current guidelines recommending PrEP only for individuals at specific risk (28–30).

For MSW and WSW, the thresholds were lower (≈0.005–0.007), suggesting that moderately transmissible bacterial STIs can circulate and that HIV transmission, although less efficient, may be sustained under particular conditions of concurrency or high prevalence. This finding is compatible with surveillance reports showing concentrated but non-negligible burdens in these groups (31, 32).

The situation is markedly different in MSM, where the thresholds were effectively null. Even infections with low per-act transmission probabilities, such as HIV or mpox, exceed this threshold and can spread efficiently. This explains why MSM populations have borne disproportionate burdens of HIV in high-income countries (33), and why rapid outbreaks of mpox in 2022–2023 spread predominantly within MSM networks (34). These results underscore the structural vulnerability of MSM networks to epidemic spread, independent of pathogen-specific factors.

### 4.2 Bridging and cross-group transmission

An implication of our analysis is the role of bisexual bridging. Although MSM networks alone can sustain epidemics, bridging fractions as small as 5%–7% suffice to propagate infections into heterosexual populations, under plausible within-group controls. This highlights a critical epidemiological mechanism: heterosexual epidemics do not require widespread risk behavior, but can emerge from relatively modest levels of bisexual mixing (33, 35). Our framework translates this into actionable prevention targets: reducing cross-group transmissibility (through PrEP, condoms, early treatment, or behavioral interventions) or lowering the effective bridge fraction can prevent spillover. The worked scenarios suggest that interventions covering only a few tens of percent of bridging partnerships may be sufficient for subcriticality, a realistic and efficient goal for prevention programs (Supplementary Material S4, S5).

### 4.3 Clinical and policy implications

For MSM, prevention strategies must acknowledge that extremely heavy-tailed degree distributions mean that a small subset of individuals contributes disproportionately to epidemic potential (36, 37). Tail-sensitive interventions, such as intensified testing, contact tracing, and event-driven PrEP targeted at high-degree individuals, offer potentially high leverage. This aligns with clinical observations that “superspreading” events in MSM communities can drive rapid incidence increases (30) (Supplementary Material S6).

For heterosexual populations, the thresholds are reassuringly positive, but the sensitivity of $T_{c}$ to extreme values cautions against complacency. In practice, a few very high-degree individuals can materially lower thresholds and facilitate sustained transmission (38). Clinically, this argues for continued attention to subgroups with high partner turnover, such as sex workers and clients, even within predominantly subcritical populations (39) (Supplementary Material S7).

The bridging framework also reframes prevention goals. Instead of universal coverage, targeted prevention along bridging edges, such as bisexual men, their partners, and networks where mixing is most likely, may yield disproportionate benefit (33, 35). Clinically, this supports inclusive risk assessments in sexual health services that do not treat heterosexual and homosexual behaviors as disjoint categories but explicitly consider bisexual partnerships (Supplementary Material S8).

### 4.4 Limitations

The limitations are intrinsic to the UK Biobank, as it comprises a non-probability cohort of volunteers aged 40–69 with healthier profiles, higher socioeconomic status, and reduced ethnic Diversity. Moreover, self-reported lifetime partner counts are subject to recall, social-desirability, and heaping biases, especially at extremes. The behavioral data lack longitudinal structure (accumulation trajectories, timing, concurrency, and turnover) and key covariates (STI history, partnership duration, and sexual practices), limiting linkage to incidence and constraining dynamic modeling. The results should therefore be interpreted as stratified internal contrasts rather than population estimates. Sensitivity analyses (RAW/EXCLUDE/EM) bracket plausible reporting behaviors; the goodness-of-fit and interval estimates used a tail-fixed bootstrap. Truncated power-law fits occasionally showed numerical degeneracy at fixed support, which is a technical, not conceptual, limitation. Finally, $T_{c}$ thresholds arise from a configuration-model approximation; concurrency and timing may increase epidemic potential relative to these static predictions Supplementary Material S9.

### 4.5 Network structure beyond degree and coupled dynamics

Our analysis uses egocentric degree data and configuration model thresholds, and therefore does not identify the underlying contact network nor capture higher-order structure (e.g., assortativity, clustering, community structure, concurrency, and temporal turnover). While degree-constrained ensembles can be generated from the observed partner-count distributions, incorporating additional structural constraints would require richer relational data and explicit network inference. In settings where partial partnership graphs or contact-tracing subnetworks are available, link-prediction methods tailored to constrained topologies may help infer missing edges and refine network-based simulations (40, 41). Finally, our framework is purely structural and does not model behavioral feedback mediated by information dynamics or attention competition, which can modulate epidemic trajectories in coupled contagion settings (42). Extending threshold-based analyses to jointly incorporate structural heterogeneity, bridging, and information-driven behavioral adaptation is a natural direction for future work.

## 5 Conclusion

We show that lifetime sexual partnership distributions differ markedly by sexual behavior stratum and translate into large gaps in empirical epidemic thresholds. Heterosexual strata exhibit heavy but finite tails with strictly positive thresholds, indicating that sustained transmission requires moderate-to-high effective per-partnership transmissibility and that control is feasible with achievable reductions in risk. In contrast, MSM networks display extreme right-tail heterogeneity and a near-zero empirical threshold, implying structural susceptibility to sustained spread even for infections with low per-partnership transmissibility (e.g., HIV) and rapid propagation for higher-transmissibility infections (e.g., mpox). We further show that modest bisexual bridging can sustain cross-group transmission under plausible within-group controls, highlighting bridging pathways as efficient prevention targets. Overall, linking tail-sensitive partnership heterogeneity to threshold estimates provides interpretable, actionable benchmarks for STI prevention and prioritization across strata.

## Data Availability

The datasets presented in this article are not readily available because access to UK Biobank data for this project was via AV under an approved application (UK Biobank Application ID: 101667). Requests to access the datasets should be directed to ukbiobank@ukbiobank.ac.uk.
