# Supplementary material for: Epidemic thresholds across sexual behavior groups in the UK Biobank
Source: Front Reprod Health. 2026 Mar 25;8:1790557. doi: 10.3389/frph.2026.1790557 (PMC13057496; doi:10.3389/frph.2026.1790557)
Supplement: Supplementary file 1 [file Table1.pdf]

Supplementary Material

Epidemic thresholds across sexual-behaviour groups in UK Biobank

Maxence Arutkin[1] and Alexandre Vallée[2,3,*]

[1]School of Chemistry, Tel Aviv University, 6997801, Tel Aviv, Israel
[2]Department of Epidemiology and Public Health, Foch Hospital, Suresnes, 92150, France
[3]IHU FOReSIGHT, Paris, France

*Corresponding Author: Alexandre Vallée, MD, PhD. Department of Epidemiology and Public Health,
Foch Hospital, Suresnes, 92150, France. Email: al.vallee@hopital-foch.com

S1 Methods

S1.1 Study design, data source, and implementation overview We conducted a cross-sectional analysis of the UK Biobank (UKB) baseline questionnaire, focusing on lifetime number of sexual partners (Data–Field 2149) in participants aged 40–69 at recruitment (2006–2010). UKB is a large volunteer cohort with consent and governance approvals in place. We emphasise internal, stratified comparisons rather than population prevalence because UKB is not sampling–representative.[1–4]

Implementation overview (discrete likelihoods, unit–cell masses, normalisation). All tail fits are discrete on $\mathbb{N}_{\geq x_{\min}}$ using identical support per stratum (top–50% tail). For the pure power law (PL),

$$P(K = k \mid \alpha) = \frac{k^{-\alpha}}{\zeta(\alpha, x_{\min})}, \qquad \ell(\alpha) = -\alpha \sum_i \log k_i - n_{\text{tail}} \log \zeta(\alpha, x_{\min}),$$

with Hurwitz zeta $\zeta(\alpha, x_{\min}) = \sum_{j=x_{\min}}^{\infty} j^{-\alpha}$. The truncated power law (TPL) uses

$$P(K = k \mid \alpha, \lambda) = \frac{k^{-\alpha} e^{-\lambda k}}{\sum_{j=x_{\min}}^{\infty} j^{-\alpha} e^{-\lambda j}}.$$

The discretised lognormal (LN) employs unit–cell masses

$$P(K = k \mid \mu, \sigma) = \frac{\Phi\Big(\frac{\log(k+\frac{1}{2})-\mu}{\sigma}\Big) - \Phi\Big(\frac{\log(k-\frac{1}{2})-\mu}{\sigma}\Big)}{\sum_{j \geq x_{\min}} \Big[\Phi\Big(\frac{\log(j+\frac{1}{2})-\mu}{\sigma}\Big) - \Phi\Big(\frac{\log(j-\frac{1}{2})-\mu}{\sigma}\Big)\Big]},$$

and stretched–exponential (SE) / exponential (EXP) models use discrete renormalisation over $k \geq x_{\min}$. Full details appear again in S1.4 and, with expanded derivations, in later

Supplement sections.

S1.2 Participants, strata, and downstream pipeline We analysed UKB baseline records with non–missing lifetime partner counts (Field 2149), sex (Field 31), the ever same–sex indicator (Field 2159), and the lifetime number of same–sex partners (Field 3669). Cleaning rules were prespecified: (i) set `same_sex_partners`=0 when `ever_same_sex`=0; (ii) when `ever_same_sex`=1 and `same_sex_partners` is missing, impute to 1; (iii) cap `same_sex_partners` at `lifetime_partners` if over–reported. We then defined four partner–sex strata: men with at least one male partner (MSM), men with only female partners (MSW), women with at least one female partner (WSW), and women with only male partners (WSM). Final counts were: WSM N = 215,734, MSW N = 177,679, MSM N = 6,428, WSW N = 5,899; total $N \simeq 405{,}740$. Analyses use baseline instance 0 only (one row per participant). Moments are computed on the sexually active subgraph ($K > 0$) unless stated otherwise.

Downstream procedures across heaping modes. All subsequent steps—fixed–support fitting, GoF bootstraps, pairwise model comparisons, and moment/threshold computations—are applied identically under each heaping mode (RAW / EXCLUDE / EM-like) described in S1.3 and implemented in S1.4–S1.6.

S1.3 Reporting bias (digit preference): prespecified sensitivity Lifetime partner counts exhibit digit preference at salient integers. We addressed this via two a priori corrections and also retained the unadjusted data:

- **RAW**: original, unaltered counts.
- **EXCLUDE**: remove observations at heap values on a prespecified grid $\{5, 10, 20, 50, 100, 200, 500, 1000, 2000, 5000, 10000\}$ (extended multiplicatively to cover the observed support).
- **EM-like**: deterministic, mass–conserving redistribution of heap mass from h into a local window $W_h = \{k : \lfloor(1-\delta)h\rfloor \le k \le \lceil(1+\delta)h\rceil,\ k \ne h\}$ with $\delta = 0.1$, using PL–like weights $w_k \propto \max(k, x_{\text{min,seed}})^{-\alpha_{\text{seed}}}$ (seeds $x_{\text{min,seed}}$=20, α_{seed}=2.0) and largest–remainder integer allocation; iterate 3 times.

All downstream fitting, bootstraps, and comparisons are identical across modes.[5–8]

S1.4 Tail definition, candidate models, and discrete likelihoods To ensure like–for–like comparisons, we define the tail per stratum and mode as the top 50% of observations and fix

$$x_{\min} = \lceil \text{quantile}_{0.50}(K) \rceil, \qquad n_{\text{tail}} = \#\{K \ge x_{\min}\},$$

requiring $n_{\text{tail}} \geq 200$; otherwise the block is skipped. All candidates are fitted by discrete maximum likelihood on the identical support $\mathbb{N}_{\geq x_{\min}}$ with no $x_{\min}$ search. Models: PL, TPL, discretised LN, SE, and EXP. Normalisation and unit–cell masses follow the expressions summarised in S1.1 (and expanded later).

S1.5 Power–law calibration and model selection (GoF, Vuong, AICc) PL GoF on fixed support. Fit the discrete PL at fixed $x_{\min}$; compute the KS distance D_{obs} (empirical vs fitted PL CCDF). Perform a parametric bootstrap with B=50,000 tails generated from the fitted PL at the same n_{tail} and $x_{\min}$; refit PL and recompute $D^{(b)}$. The bootstrap p is $\hat{p} = \frac{1}{B}\sum_b \mathbf{1}\{D^{(b)} \geq D_{\text{obs}}\}$. We report 99% Clopper–Pearson intervals $[p_\ell, p_u]$ and decide at threshold $\tau = 0.10$: reject if $p_u < \tau$; accept if $p_\ell > \tau$; indeterminate otherwise. In our outputs, every stratum–mode block triggered `tpl_degenerate` ($\hat{\lambda} \approx 0$), so the nested LR and information criteria for TPL were not interpreted.

Pairwise comparisons on the same tail. Alternatives (LN, SE, EXP) vs PL use Vuong's Z (non–nested). TPL vs PL uses the nested LR test. We also compute

$$\text{AICc} = -2\log L + 2k + \frac{2k(k+1)}{n_{\text{tail}} - k - 1}, \qquad \Delta\text{AICc}_{\text{alt}-\text{PL}} = \text{AICc}_{\text{alt}} - \text{AICc}_{\text{PL}},$$

with parameter counts $k = 1$ (PL/EXP) and $k = 2$ (LN/TPL/SE).

PL parameter uncertainty and TPL guard. A 99% CI for α is obtained via a tail–fixed nonparametric bootstrap (B=50,000) when the in–house routine fails. TPL fits are flagged as degenerate near $\lambda \downarrow 0$ or when normalisation is ill–conditioned; such blocks are excluded from pairwise interpretation.

S1.6 Moments, thresholds, and per–partnership mapping The configuration–model critical bond transmissibility is

$$T_c \;=\; \frac{\mathbb{E}[K]}{\mathbb{E}[K^2] - \mathbb{E}[K]}\,, \tag{S1}$$

so $T_c = 0$ iff $\mathbb{E}[K^2] = \infty$.[9,10] For the main results we estimate moments empirically from the observed counts (per stratum and heaping mode):

$$\hat{m}_1 = \frac{1}{N}\sum_i K_i, \quad \hat{m}_2 = \frac{1}{N}\sum_i K_i^2, \quad \hat{\kappa} = \frac{\hat{m}_2 - \hat{m}_1}{\hat{m}_1}, \quad \hat{T}_c = \hat{\kappa}^{-1}.$$

This avoids model–implied moment bias and ensures T_c is a direct finite–population functional. We set $\kappa \equiv (m_2 - m_1)/m_1$ and use per–stratum estimates $\hat{\kappa}$ to parameterise κ_A, κ_B in S1.7. (Hybrid head+tail or model–implied moments can be reported; conclusions are unchanged.)

Per–partnership transmissibility. If per–act risk is p over n acts, the per–partnership transmission probability is $T_{\text{edge}} = 1 - (1-p)^n$. This mapping is used only to translate T_c into coverage targets in the Discussion.

S1.7 Two–group bridge threshold, coverage, and elasticities Let A (MSM) and B (WSM) with next–generation matrix

$$G = \begin{pmatrix} a & b \\ c & d \end{pmatrix}, \quad a = \kappa_A T_{AA}(1-\phi),\ \ b = \kappa_A T_{AB}\phi,\ \ c = \kappa_B T_{BA}\psi,\ \ d = \kappa_B T_{BB}(1-\psi),$$

where κ are configuration factors from S1.6, T_{ij} are per–edge transmissibilities, and ϕ (MSM$\to$WSM), ψ (WSM$\to$MSM) are fractions of cross–group edges. Under degree-independent mixing, ϕ and ψ are linked by edge-balance (the implied number of cross-group edges should match when counted from each group); we therefore treat (ϕ, ψ) as illustrative mixing parameters in the worked thresholds. If $a < 1$ and $d < 1$, then

$$\rho(G) > 1 \iff \kappa_A \kappa_B\, T_{AB} T_{BA}\, \phi\psi > (1-a)(1-d).$$

Symmetric bridge share. If $\phi = \psi = \theta$,

$$\theta_{\min} = \sqrt{\frac{(1-a)(1-d)}{\kappa_A \kappa_B\, T_{AB} T_{BA}}}\,.$$

Asymmetric targeting. For fixed $\phi = \phi_0$,

$$\psi_{\min}(\phi_0) = \frac{(1-a)(1-d)}{\kappa_A \kappa_B\, T_{AB} T_{BA}\, \phi_0}\,.$$

Coverage on bridges (reducing T_{AB}, T_{BA}). If prevention reduces cross–group transmissibilities by $t \in (0,1]$ (so $T_{AB} \to tT_{AB}$ and $T_{BA} \to tT_{BA}$), then the minimum reduction at given ϕ, ψ is

$$t_{\min} = \sqrt{\frac{(1-a)(1-d)}{\kappa_A \kappa_B\, T_{AB}^{(0)} T_{BA}^{(0)}\, \phi\psi}}\,.$$

With intervention effectiveness e and coverage c, the effective reduction is $t = 1 - ec$ and the minimum coverage is $c_{\min} = (1 - t_{\min})/e$.

Elasticities: from the symmetric threshold,

$$\mathrm{d}\ln\theta_{\min} = \tfrac{1}{2}[\mathrm{d}\ln(1-a) + \mathrm{d}\ln(1-d) - \mathrm{d}\ln\kappa_A - \mathrm{d}\ln\kappa_B - \mathrm{d}\ln T_{AB} - \mathrm{d}\ln T_{BA}]\,.$$

Halving $\theta_{\min}$ requires a fourfold improvement in $\kappa_A T_{AB} \cdot \kappa_B T_{BA}$ or large changes in $(1-a)$ and $(1-d)$. Full derivations and sensitivities are provided in supp:S8.

S1.8 Prespecification, computation, and sensitivity All hyperparameters were fixed a priori: tail keep proportion 50%; minimum tail size $n_{\mathrm{tail}} \geq 200$; GoF and CI bootstraps B=50,000 at 99% CL; decision threshold τ=0.10; EM window δ=0.1 and 3 iterations; heap grid as above. Computations use `powerlaw` for discrete fits and bootstraps,[11] multiprocessing with per–process single–threaded BLAS, and fixed seeds for reproducibility. Sensitivity to heaping (RAW/EXCLUDE/EM), to record values (remove/double the sample maximum), and to finite–population domain size are reported alongside the main results.

supp:S2

S2. Discrete tail likelihoods and support

This section expands S1.1 and S1.4. Notation matches S1: the tail support is discrete on $\mathbb{N}_{\geq x_{\min}}$ with $x_{\min}$ fixed at the stratum–specific median (top–50% tail), and all likelihoods are fitted on the identical support with no $x_{\min}$ search. Primary results use empirical moments (S1.6); the empirical–head + model–tail splice is used only for the optional hybrid check in supp:S5.

Discrete support and head handling. All tail models are defined on $k \in \{x_{\min}, x_{\min}{+}1, \ldots\}$. Below $x_{\min}$, the empirical pmf is retained only when constructing the hybrid head+tail pmf in supp:S5. It is not used for the main (empirical–moment) analyses.

Pure power law (PL). For $\alpha > 1$,

$$P(K = k \mid \alpha) = \frac{k^{-\alpha}}{\zeta(\alpha, x_{\min})}, \qquad \ell(\alpha) = \sum_{i=1}^{n_{\mathrm{tail}}} \log P(k_i \mid \alpha) = -\alpha \textstyle\sum_i \log k_i - n_{\mathrm{tail}} \log \zeta(\alpha, x_{\min}),$$

where $\zeta(\alpha, x_{\min}) = \sum_{j=x_{\min}}^{\infty} j^{-\alpha}$ is the Hurwitz zeta normaliser.

Truncated power law (TPL). For $\alpha > 1$, $\lambda \geq 0$,

$$P(K = k \mid \alpha, \lambda) = \frac{k^{-\alpha} e^{-\lambda k}}{Z_{\mathrm{TPL}}(\alpha, \lambda, x_{\min})}, \qquad Z_{\mathrm{TPL}}(\alpha, \lambda, x_{\min}) = \sum_{j=x_{\min}}^{\infty} j^{-\alpha} e^{-\lambda j}.$$

Near $\lambda \downarrow 0$ numerical degeneracy can occur (guard and policy in S1.5).

Discretised lognormal (LN). Let $Y = \log K$ with $Y \sim \mathcal{N}(\mu, \sigma^2)$ and $\sigma > 0$. Using unit–cell masses,

$$P(K = k \mid \mu, \sigma) = \frac{\Phi\!\left(\frac{\log(k+\frac{1}{2})-\mu}{\sigma}\right) - \Phi\!\left(\frac{\log(k-\frac{1}{2})-\mu}{\sigma}\right)}{\sum_{j=x_{\min}}^{\infty} \left[\Phi\!\left(\frac{\log(j+\frac{1}{2})-\mu}{\sigma}\right) - \Phi\!\left(\frac{\log(j-\frac{1}{2})-\mu}{\sigma}\right)\right]}, \quad k \geq x_{\min}.$$

Stretched exponential (SE) and exponential (EXP). For $\beta \in (0, 1]$, $\lambda > 0$,

$$\text{SE: } P(K = k \mid \lambda, \beta) \;\propto\; k^{\beta-1} e^{-\lambda k^{\beta}}, \qquad \text{EXP: } P(K = k \mid \lambda) \;\propto\; e^{-\lambda k},$$

with discrete renormalisation $\sum_{k \geq x_{\min}} P(K = k) = 1$ on the identical support.

Fixed support. All models are fitted by maximum likelihood on the fixed top–50% tail: $x_{\min} = \lceil \text{quantile}_{0.50}(K) \rceil$, $n_{\text{tail}} = \#\{K \geq x_{\min}\}$ (require $n_{\text{tail}} \geq 200$). This ensures like–for–like comparisons across PL, TPL, LN, SE, and EXP (see calibration and selection in S1.5).

supp:S3

S3. Heaping correction (RAW / EXCLUDE / EM-like)

Scope and role. Digit preference at salient integers (e.g., $5, 10, 20, 50, 100, \ldots$) is addressed via two prespecified sensitivity modes in addition to the primary **RAW** analysis. All downstream steps (fixed-support tail fits, GoF bootstraps, pairwise comparisons, and empirical moment/threshold computation) are applied identically across modes (cf. S1.4–S1.6). The fixed top–50% tail threshold $x_{\min}$ (the stratum-specific median) is recomputed within each mode before fitting.

Heaps and grid. Let the heaping grid be

$$\mathcal{H} = \{5, 10, 20, 50, 100, 200, 500, 1000, 2000, 5000, 10000\} \cap [1, K_{\max}],$$

extended multiplicatively to cover the observed support. The grid does not include 0; counts at $K = 0$ are never altered.

Modes.

- **RAW (primary):** Use the original, unaltered counts.
- **EXCLUDE (sensitivity):** Remove observations with $K \in \mathcal{H}$; retain all others unchanged.
- **EM-like (sensitivity, deterministic, mass-conserving):** For each $h \in \mathcal{H}$ (processed in ascending order), let

  $$W_h = \{k : \lfloor (1-\delta)h \rfloor \leq k \leq \lceil (1+\delta)h \rceil, \; k \neq h\}, \quad \delta = 0.1.$$

  Intersect W_h with the observed support $[1, K_{\max}]$. Let n_h be the number of observations

at $K = h$. Define weights

$$w_k \;\propto\; \max(k, x_{\min,\text{seed}})^{-\alpha_{\text{seed}}}, \quad x_{\min,\text{seed}} = 20,\ \alpha_{\text{seed}} = 2.0,$$

normalise $\sum_{k \in W_h} w_k = 1$, and deterministically allocate n_h counts to $\{k \in W_h\}$ by largest-remainder rounding of $n_h\, w_k$. Apply this sweep to all $h \in \mathcal{H}$ and iterate the full grid three times. No random seeds are used.

Invariants and safeguards. The EM-like procedure (i) conserves total sample size and total mass, (ii) preserves support bounds ($K \in [1, K_{\max}]$), (iii) leaves $K = 0$ unchanged, (iv) maintains a monotone empirical CDF/CCDF, and (v) is local (mass from h is redistributed only within W_h). Because redistribution is integer-valued and local, the observed median (and thus $x_{\min}$) can move by at most a few units; we recompute $x_{\min}$ per mode before fitting to ensure identical-support comparisons within each mode (S1.4).

Downstream processing (unchanged across modes). After applying RAW / EXCLUDE / EM-like, we: (i) recompute the stratum-specific median to set $x_{\min}$ (top–50% tail); (ii) fit all discrete tail candidates on the identical support $\mathbb{N}_{\geq x_{\min}}$ by maximum likelihood; (iii) run the PL GoF parametric bootstrap and pairwise model selection (Vuong, ΔAICc) as in S1.5; and (iv) compute empirical moments and T_c as in S1.6. Per–mode sample sizes N (overall and tail) are reported in the main tables.

Notes. (1) "EM-like" denotes a deterministic local reallocation scheme; it is not an expectation–maximisation estimator. The hyperparameters $(\delta, x_{\min,\text{seed}}, \alpha_{\text{seed}})$ are prespecified and fixed across strata and modes. (2) Heaping correction is used only for sensitivity; the primary inferences are reported under RAW. (3) Because all tail fits are on fixed, identical support within a mode, model comparisons are not confounded by $x_{\min}$ variation (S1.4–S1.5).

supp:S4

S4. Calibration and model selection

Scope and alignment. This section expands S1.5. All goodness-of-fit (GoF) and model-comparison procedures are conducted on the fixed top–50% tail (per stratum and mode), with identical discrete support $\{k \in \mathbb{N} : k \geq x_{\min}\}$ and no $x_{\min}$ search (see S1.4). Primary inference is reported under **RAW**; **EXCLUDE** and **EM-like** are sensitivity modes (S3).

Power–law GoF (parametric bootstrap at fixed support). For each stratum–mode tail:

(i) Fit the discrete power law (PL) on $\{k \geq x_{\min}\}$ by MLE; let $\hat{\alpha}$ be the exponent.

(ii) Compute the (discrete) KS distance D_{obs} between the empirical and fitted PL tail distributions (CCDF/CDF are equivalent under KS; we use the CCDF for diagnostics).

(iii) Generate $B = 50{,}000$ bootstrap samples of size n_{tail} from $\text{PL}(\hat{\alpha}; x_{\min})$ on $\mathbb{N}_{\geq x_{\min}}$.

(iv) For each bootstrap sample, refit PL at the same $x_{\min}$ to obtain $D^{(b)}$.

(v) Estimate $\hat{p} = \frac{1}{B}\sum_b \mathbf{1}\{D^{(b)} \geq D_{\text{obs}}\}$ and report the exact 99% Clopper–Pearson interval $[p_\ell, p_u]$.

Decision rule (prespecified threshold $\tau = 0.10$): reject PL if $p_u < \tau$; accept PL if $p_\ell > \tau$; indeterminate otherwise. With $B = 50{,}000$, the Monte Carlo s.e. of $\hat{p}$ is $\leq \sqrt{\hat{p}(1-\hat{p})/B}$, which is < 0.005 when $\hat{p} \in [0.05, 0.95]$.

Pairwise model comparison on identical support. Let $\ell_{\text{alt}}(k)$ and $\ell_{\text{PL}}(k)$ denote pointwise log pmf values on the tail for a candidate model alt and PL, respectively. Define per-observation log-likelihood ratios

$$d_i = \ell_{\text{alt}}(k_i) - \ell_{\text{PL}}(k_i), \qquad \bar{d} = \frac{1}{n_{\text{tail}}}\sum_{i=1}^{n_{\text{tail}}} d_i, \qquad s_d^2 = \frac{1}{n_{\text{tail}}-1}\sum_{i=1}^{n_{\text{tail}}}(d_i - \bar{d})^2.$$

Vuong's statistic (non-nested pairs LN, SE, EXP vs PL) is

$$Z = \frac{\sqrt{n_{\text{tail}}}\,\bar{d}}{s_d},$$

reported with a two-sided normal p; $Z > 0$ favours alt, $Z < 0$ favours PL.

For information criteria we compute

$$\text{AICc} = -2\log L + 2k + \frac{2k(k+1)}{n_{\text{tail}} - k - 1}, \qquad \Delta\text{AICc}_{\text{alt}-\text{PL}} = \text{AICc}_{\text{alt}} - \text{AICc}_{\text{PL}},$$

with parameter counts $k = 1$ (PL, EXP) and $k = 2$ (LN, SE, TPL). Negative ΔAICc favours alt. Both Vuong Z and ΔAICc are computed on the same tail support to ensure like-for-like comparison (S1.4).

Nested comparison: TPL vs PL and degeneracy policy. For TPL vs PL (nested), we use the nested LR test as implemented in the fitting code, with the caveat that TPL can be numerically degenerate at or near the boundary $\lambda \downarrow 0$ under fixed $x_{\min}$. Degenerate TPL fits are flagged and excluded from pairwise interpretation (policy as in S1.5).

Table S1: Pairwise comparisons on identical support (RAW mode): alternative models vs power law (PL). x_{comp} and n_{comp} denote the common tail support and size. Vuong's Z and ΔAICc are shown for non–nested models (negative ΔAICc favors the alternative). All TPL rows are marked "—" because the TPL MLE hit the boundary ($\hat{\lambda} \to 0$) on the fixed tail in 12/12 blocks (see S1.5).

Stratum	Alt model	x_{comp}	n_{comp}	Z	ΔAICc	Decision
MSM	EXP	12	3,236	-7.950	16205.70	PL better
MSM	LN	12	3,236	-5.864	414.30	PL better
MSM	SE	12	3,236	-6.902	803.30	PL better
MSM	TPL	12	3,236	–	–	—
MSW	EXP	4	94,919	-15.098	54285.28	PL better
MSW	LN	4	94,919	42.072	-6572.55	ALT better
MSW	SE	4	94,919	-0.907	3082.08	PL better
MSW	TPL	4	94,919	–	–	—
WSM	EXP	3	111,407	-6.664	8328.94	PL better
WSM	LN	3	111,407	53.208	-11365.74	ALT better
WSM	SE	3	111,407	0.395	-1344.32	ALT better
WSM	TPL	3	111,407	–	–	—
WSW	EXP	8	3,214	-2.009	1002.75	PL better
WSW	LN	8	3,214	9.945	-236.64	ALT better
WSW	SE	8	3,214	5.356	-242.79	ALT better
WSW	TPL	8	3,214	–	–	—

Uncertainty for PL exponent. When the in-house bootstrap is unstable at fixed $x_{\min}$, we use a tail-fixed nonparametric bootstrap: resample the observed tail with replacement $B = 50{,}000$ times, refit PL at the same $x_{\min}$, and report percentile 99% CIs for α.

Reporting and decision cascade. For each stratum–mode we report: (B, hits, $\hat{p}$, 99% CI, GoF decision) for PL; and, for each alt∈{LN, SE, EXP, TPL}, the Vuong Z (or nested LR for TPL) and ΔAICc. GoF acceptance of PL does not imply optimality; model ranking relies on pairwise comparisons on identical support.

Numerical safeguards. All likelihoods are evaluated on integers $k \geq x_{\min}$; LN cell masses use stable log-CDF differences; normalisations are computed in log-space where needed. Tail blocks with $n_{\text{tail}} < 200$ are skipped by design (S1.4). RNG seeds, BLAS settings, and reproducibility protocol follow S1.8.

supp:S5

S5. Hybrid moments (optional sensitivity, not used in primary results)

Purpose. Primary thresholds in the MAIN use empirical moments (S1.6). This section defines an optional head–empirical / tail–model splice to check robustness of κ and T_c

to replacing the empirical tail by a fitted tail distribution. Results from this hybrid construction are sensitivity only.

Hybrid pmf. Let $\hat{p}_{\text{emp}}(k)$ be the empirical pmf for the head $k < x_{\min}$, and let $p_{\text{tail}}(k \mid \hat{\theta})$ be any fitted discrete tail pmf on $\{k \geq x_{\min}\}$ (PL, TPL, LN, SE, EXP as defined in S2), each fitted on the identical support (S1.4). With empirical tail mass

$$\pi_{\text{tail}} = \sum_{k \geq x_{\min}} \hat{p}_{\text{emp}}(k),$$

define the hybrid pmf

$$\tilde{p}(k) = \begin{cases} \hat{p}_{\text{emp}}(k), & k < x_{\min}, \\ \pi_{\text{tail}} \dfrac{p_{\text{tail}}(k \mid \hat{\theta})}{\sum_{j \geq x_{\min}} p_{\text{tail}}(j \mid \hat{\theta})}, & k \geq x_{\min}. \end{cases}$$

Hybrid moments and threshold. Compute

$$m_1 = \sum_k k\, \tilde{p}(k), \qquad m_2 = \sum_k k^2\, \tilde{p}(k), \qquad \kappa = \frac{m_2 - m_1}{m_1}, \qquad T_c = \kappa^{-1}.$$

These are reported only as sensitivity checks; the MAIN relies on empirical moments (S1.6).

Uncertainty (optional). CIs for hybrid functionals can be obtained by a tail-fixed bootstrap that resamples the head empirically and refits the tail on the fixed support, or by parametric draws from $p_{\text{tail}}(\cdot \mid \hat{\theta})$; both keep $x_{\min}$ fixed (S1.4).
supp:S6

S6. Extreme values (records): diagnostics and sensitivity

Exact one-record update (finite-sample sensitivity). Let $m_1 = \frac{1}{N}\sum_i K_i$ and $m_2 = \frac{1}{N}\sum_i K_i^2$ be the empirical moments used in S1.6. If a single observation is changed $k \mapsto k'$ in a sample of size N, then

$$m_1' = m_1 + \frac{k' - k}{N}, \qquad m_2' = m_2 + \frac{k'^2 - k^2}{N}, \qquad T_c' = \frac{m_1'}{m_2' - m_1'}.$$

Hence T_c is quadratically sensitive to large k through $k'^2 - k^2$, which is why observed records can materially perturb T_c in finite samples.

Tail-dependent predictions for maxima. Let $K_{(N)} = \max\{K_1, \ldots, K_N\}$ and $n_{\text{tail}} = \#\{K \geq x_{\min}\}$.

Discretised lognormal (LN). If K follows a discretised lognormal tail with latent $\log K \sim \mathcal{N}(\mu, \sigma^2)$ (S2), a standard quantile approximation gives

$$K_{(N)} \;\approx\; \exp\Bigl(\mu + \sigma\,\Phi^{-1}\bigl(1 - \tfrac{1}{N}\bigr)\Bigr),$$

i.e., maxima grow sub-polynomially with N (finite variance; "tame" records).

Pure power law (PL). If K follows a discrete PL on $\{k \geq x_{\min}\}$ with exponent $\alpha > 1$, the tail of size n_{tail} yields

$$K_{(n_{\text{tail}})} \;\approx\; x_{\min}\, n_{\text{tail}}^{1/(\alpha-1)}.$$

For $\alpha \leq 3$, model-implied second moments diverge asymptotically and predicted maxima can be astronomical relative to observed records—an empirical signal for truncation/mixture in real data even when mid-tails appear PL-like on log–log axes.

Observed records and contribution to m_2 (RAW, descriptive). For transparency, we report the observed RAW records per stratum and their share of m_2. These quantities explain why T_c shifts under exact removal/doubling of a single record (via the update above). Numeric values and exact T_c recomputations are provided in the MAIN tables or inlined in the Results where noted; when omitted from the MAIN for brevity, they remain available upon request using the same deterministic recomputation.

supp:S7

S7. Per–partnership transmissibility T_{edge} and coverage mapping (interpretive only)

Purpose. This section is interpretive. Primary thresholds in the MAIN use empirical moments and T_c as defined in supp:S1 (S1.6). Here we translate a partnership-level transmissibility into minimal coverage targets. No estimation in the paper depends on S7.

Partnership transmissibility. For a partnership with n acts of type with per-act risk p,

$$T_{\text{edge}} \;=\; 1 - (1-p)^n.$$

If partnerships are heterogeneous in (p, n) with joint distribution $\mathcal{D}$, a scalar summary can be formed as

$$T^{\star}_{\text{edge}} \;=\; \mathbb{E}_{\mathcal{D}}[\,1 - (1-p)^n\,]\,,$$

and the mappings below apply with $T^{\star}_{\text{edge}}$ in place of T_{edge}.

Coverage mapping (single multiplicative intervention). Let an intervention of effectiveness $e \in (0,1]$ cover a fraction $c \in [0,1]$ of partnerships (or persons) relevant to transmission. If its effect is multiplicative on partnership risk, the effective transmissibility is

$$T'_{\text{edge}} \;=\; (1 - ec)\, T_{\text{edge}}.$$

Subcriticality on a configuration network requires $T'_{\text{edge}} < T_c$ (with T_c from S1.6), yielding the minimum coverage

$$c_{\min} \;=\; \frac{1 - T_c/T_{\text{edge}}}{e} \quad \text{clipped to } [0,1].$$

Notes. (i) If multiple independent multiplicative interventions apply, replace $1 - ec$ by $\prod_j (1 - e_j c_j)$. (ii) This mapping is monotone in T_{edge} and e; it is used only to interpret T_c in clinical terms and does not alter any primary estimates.

supp:S8

S8. Two–group bridge model: guardrail, derivation, and sensitivities

Setup (matches S1.7). Let groups A (e.g., MSM) and B (e.g., WSM) have a next–generation matrix

$$G = \begin{pmatrix} a & b \\ c & d \end{pmatrix}, \qquad \begin{array}{ll} a = \kappa_A\, T_{AA}\,(1-\phi), & b = \kappa_A\, T_{AB}\,\phi, \\ c = \kappa_B\, T_{BA}\,\psi, & d = \kappa_B\, T_{BB}\,(1-\psi), \end{array}$$

where $\kappa_{A/B} = (m_2 - m_1)/m_1$ come from empirical moments (S1.6), T_{ij} are per–edge transmissibilities, and $\phi, \psi \in [0,1]$ are cross–group edge fractions (A→B and B→A, respectively).

Guardrail (equivalent threshold condition). If each group is subcritical in isolation ($a < 1$, $d < 1$), then

$$\rho(G) > 1 \iff \kappa_A \kappa_B\, T_{AB} T_{BA}\, \phi\psi \;>\; (1-a)(1-d)\,.$$

Derivation. For a 2×2 nonnegative G, $\rho(G) > 1$ iff $\det(I - G) < 0$. Here $\det(I - G) = (1-a)(1-d) - bc$ with $b = \kappa_A T_{AB} \phi$ and $c = \kappa_B T_{BA} \psi$.

Symmetric bridge share. If $\phi = \psi = \theta$,

$$\theta_{\min} = \sqrt{\frac{(1-a)(1-d)}{\kappa_A \kappa_B\, T_{AB} T_{BA}}}.$$

Asymmetric bridge share. Fix $\phi = \phi_0 > 0$. The minimum ψ is

$$\psi_{\min}(\phi_0) = \frac{(1-a)(1-d)}{\kappa_A \kappa_B \, T_{AB} T_{BA} \, \phi_0}.$$

Reducing cross–group transmissibility on bridges. If prevention on bridge edges scales T_{AB}, T_{BA} by $t \in (0,1]$ (e.g., PrEP, condoms, TasP) then

$$t_{\min} = \sqrt{\frac{(1-a)(1-d)}{\kappa_A \kappa_B \, T_{AB}^{(0)} T_{BA}^{(0)} \, \phi \psi}},$$

with $T_{ij}^{(0)}$ the baseline values. If a single intervention of effectiveness e covers a fraction c of bridges, $t = 1 - ec$ and the minimum coverage is $c_{\min} = (1 - t_{\min})/e$.

Elasticities (diagnostic). From the symmetric threshold,

$$\mathrm{d}\ln \theta_{\min} = \tfrac{1}{2}\Big[\mathrm{d}\ln(1-a) + \mathrm{d}\ln(1-d) - \mathrm{d}\ln \kappa_A - \mathrm{d}\ln \kappa_B - \mathrm{d}\ln T_{AB} - \mathrm{d}\ln T_{BA}\Big],$$

so $\theta_{\min}$ is square–root sensitive to multiplicative improvements in within–group control ($1-a$, $1-d$), dispersion (κ), and cross–group transmissibility (T_{AB}, T_{BA}).

Scope and usage. S8 provides the derivations underlying the bridge claims in the MAIN; it uses $\kappa_{A/B}$ from empirical moments (S1.6) and does not introduce additional modelling assumptions. Worked numeric examples, if shown, appear in the MAIN Results; none are required here.

supp:S9

S9. Computation, reproducibility, and numerical safeguards

Software and environment. All fits and bootstraps use `powerlaw` with the discrete option on fixed support (S1.4–S1.5). Multiprocessing uses per–process single-threaded BLAS; the EM-like heaping adjustment (S3) is deterministic. Primary inference is under **RAW**; **EXCLUDE** and **EM-like** are sensitivity modes (S3).

Determinism and seeds. Random components (parametric PL GoF bootstrap; tail-fixed nonparametric bootstrap for α CIs) use fixed seeds per stratum–mode with B = 50,000 replicates (S1.5). The EM-like redistribution is seed-free and exactly reproducible.

Fixed support and skip rule. For each stratum–mode, the tail is the top 50% of observed counts; $x_{\min}$ is the stratum–mode median (S1.4). All candidate tails are fitted and compared on the identical discrete support $\{k \geq x_{\min}\}$. Tail blocks with $n_{\text{tail}} < 200$ are skipped (S1.4).

Numerical safeguards. (i) Likelihoods on integers $k \geq x_{\min}$; (ii) discretised lognormal cell masses via stable log–CDF differences; (iii) normalisers (Hurwitz zeta for PL; Z_{TPL}) guarded with log-space evaluation; (iv) TPL boundary fits flagged `tpl_degenerate` and excluded from pairwise interpretation (S1.5); (v) no special handling for maxima—record sensitivity is treated analytically in S6.

Dynamic considerations (scope). All thresholds in the MAIN use the static configuration-model formula (S1.6). Timing and concurrency can shift epidemic potential; this is acknowledged as a limitation in the MAIN. No dynamic modelling is performed; S7–S8 provide interpretive mappings and bridge guardrails only.

References

[1] Anne M. Johnson et al. Sexual behaviour in britain: Partnerships, practices, and hiv risk behaviours (natsal-3). *The Lancet*, 382(9907):1830–1844, 2013. doi: 10.1016/S0140-6736(13)61947-7.

[2] Bob Erens et al. Methodology of the third british national survey of sexual attitudes and lifestyles (natsal-3). *BMJ Open*, 3(10):e003593, 2013. doi: 10.1136/bmjopen-2013-003593.

[3] Catherine H. Mercer et al. Changes in sexual attitudes and lifestyles in britain through the life course and over time: Findings from natsal-3. *The Lancet*, 382(9907):1781–1794, 2013. doi: 10.1016/S0140-6736(13)62035-8.

[4] Deven T. Hamilton, Mark S. Handcock, and Martina Morris. Degree distributions in sexual networks: A framework for evaluating evidence. *Sexually Transmitted Diseases*, 35(1):30–40, 2008. doi: 10.1097/OLQ.0b013e3181453a84.

[5] Daniel F. Heitjan and Donald B. Rubin. Ignorability and coarse data. *The Annals of Statistics*, 19(4):2244–2253, 1991. doi: 10.1214/aos/1176348396.

[6] Xiaoyan Wang and Daniel F. Heitjan. Modeling heaping in self-reported cigarette counts. *The Annals of Applied Statistics*, 2(4):1247–1265, 2008. doi: 10.1214/08-AOAS181.

[7] Forrest W. Crawford, Robert E. Weiss, and Marc A. Suchard. Sex, lies and self-reported counts: Bayesian mixture models for heaping in longitudinal count data via birth–death processes. *The Annals of Applied Statistics*, 9(2):572–596, 2015. doi: 10.1214/15-AOAS812.

[8] Haim Y. Bar and Dean R. Lillard. Accounting for heaping in retrospectively reported event data: A mixture-model approach. *Statistics in Medicine*, 31(27):3347–3365, 2012. doi: 10.1002/sim.5419.

[9] Michael Molloy and Bruce Reed. A critical point for random graphs with a given degree sequence. *Random Structures & Algorithms*, 6(2-3):161–180, 1995. doi: 10.1002/rsa.3240060204.

[10] M. E. J. Newman. Spread of epidemic disease on networks. *Physical Review E*, 66(1):016128, 2002. doi: 10.1103/PhysRevE.66.016128.

[11] Jeff Alstott, Ed Bullmore, and Dietmar Plenz. powerlaw: A python package for analysis of heavy-tailed distributions. *PLOS ONE*, 9(1):e85777, 2014. doi: 10.1371/journal.pone.0085777.
